# Supplementary figures and images for: Culture-independent detection and characterisation of Mycobacterium tuberculosis and M. africanum in sputum samples using shotgun metagenomics on a benchtop sequencer
Source: PeerJ. 2014 Sep 23;2:e585. doi: 10.7717/peerj.585 (PMC4179564; doi:10.7717/peerj.585)

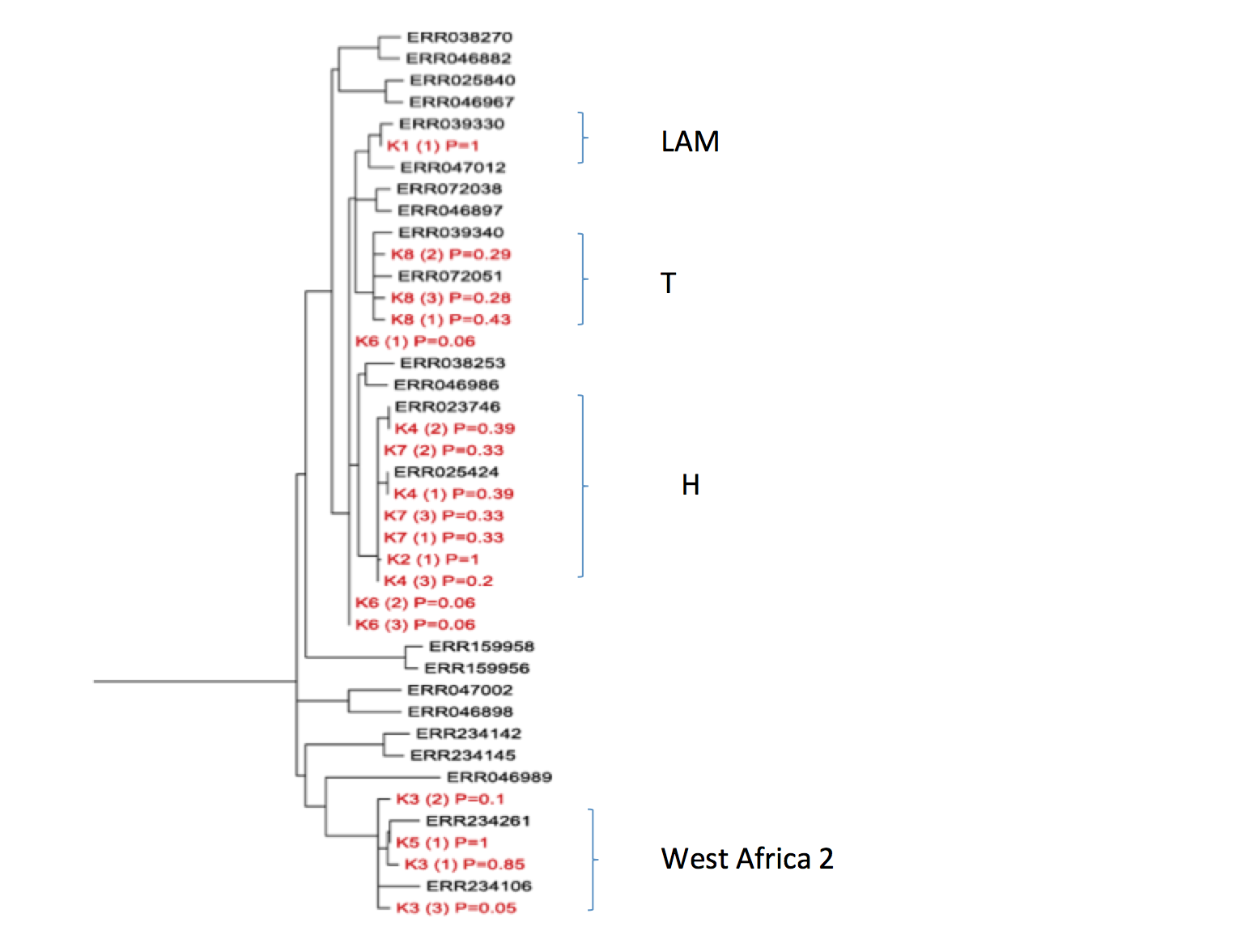

Supplement: Figure S1 — For each sample, the majority bases at each reference SNP position (or gaps if there was no coverage at that position) were concatenated and the sequence was placed in the reference tree using pplacer (see Methods). The output from pplacer (jplace file) was parsed and a new file produced, such that each alternative placement for a sample could be displayed in the tree along with the posterior probability of that placement. Trees were generated from the modified place file, using guppy from the pplacer suite of programs. Only the top 3 placements for each sample are shown. The combined pp values for each alternative placement of sample in a clade were used to ascertain the likelihood of that sample belonging to that clade. [file peerj-02-585-s001.png]
